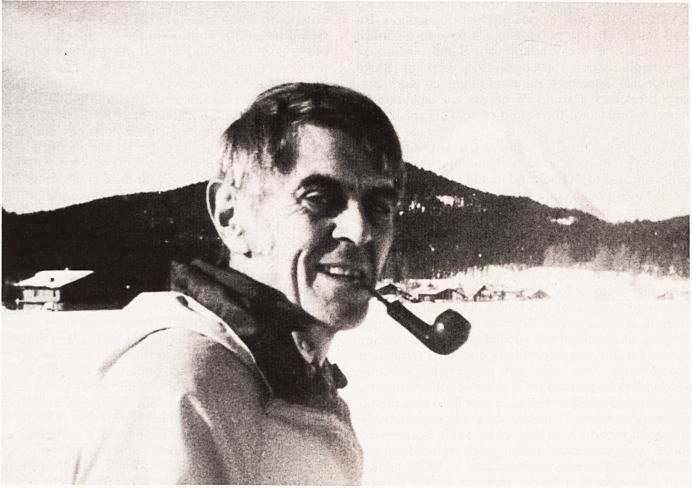# Dr. Colin Tribe

**Published:** 1984-04

**Authors:** 


### Obituary

### Dr Colin Tribe M.A., D.M., B.Ch., F.R.C.Path., D.T.M. & H.

Dr Colin Tribe, Consultant Histopathologist at Southmead Hospital, Bristol, Clinical Teacher in Pathology in the University of Bristol and Assistant Editor of this *Journal* died suddenly on 26th December 1983 aged 55 years.

Colin Richard Tribe was born in London, the only son of Sir Frank Tribe, a distinguished civil servant. Like his father and grandfather before him and also his own three sons he was educated at Clifton College. At Trinity College, Oxford he gained B.A. in animal physiology in 1950, proceeding to M.A. in 1954. His clinical training was received at Middlesex Hospital, London and he qualified with B.M., B.Ch. (Oxon.) in 1953. After house appointments in Ashford, Middlesex he took a short service commission 1955–59 in the Royal Army Medical Corps where he was trained in pathology and served as a junior specialist for two years in Singapore. He also commanded the blood transfusion unit in the Suez expedition in 1956. On leaving the army he returned to the Middlesex Hospital as Assistant Pathologist in the Bland-Sutton Institute and was seconded to Stoke Mandeville Hospital. During his four years there as registrar and senior registrar he not only began to take a life-long interest in rheumatic diseases but, with his flair for making the utmost use of whatever material came to hand, made a special study of the pathology of paraplegic patients which formed the basis of the thesis which gained him his D.M.(Oxon.) in 1964. It also led to his fascination with amyloid disease on which he was later to become a world authority. In the same year he became a Member of the Royal College of Pathologists, being elected to the Fellowship in 1974.

After a period of study in the U.S.A. as Fellow in Pathology at Johns Hopkins Hospital, Baltimore, working on renal disease with Robert Heptinstall, he was appointed Consultant Pathologist at High

Wycombe where the hospital was destined to undergo rapid expansion. Over the next six years he not only supervised the transformation of a small laboratory where he was the only pathologist with six technicians into a fully fledged department with four consultants and a technical staff of thirty, but threw himself wholeheartedly into every aspect of the widening life of the rapidly growing hospital, taking a particular interest in postgraduate education. He also found time to publish in 1969, with a clinical colleague, his monogrraph on Renal Failure in Paraplegia which, based on his work at Stoke Mandeville, remains a standard reference work, and also produced a string of papers on amyloidosis, renal failure and the rapid diagnosis of breast tumours.

Many of us, having achieved a sparkling new laboratory, newly staffed and thriving, might have been tempted to sit back and regard our major task in life as having been completed, but not Colin! He felt the need to move to a larger department where he would not be the only histopathologist but would have colleagues with whom to discuss problems, where he would have junior staff to train and students to teach and which would generate more abundant material for his fertile brain to study. It so happened that just at this time a new post had been created at Southmead Hospital with the principal purpose of developing a specialised histopathology service for the new nephrology and renal transplant departments. This was just the challenge Colin needed and he applied for and was appointed to the position. Sadly before he could take it up he suffered a heart attack and characteristically and generously offered to withdraw so that, as he put it, Southmead could have someone with a more assured future. Wisely this offer was declined and in 1971, apparently fully recovered, he commenced work in Bristol.

Never could the man and the job been better suited. The next thirteen years saw the fruits of the seeds sown in his early studies of renal failure and amyloid disease. He set himself to learn the new techniques of electron microscopy and immunofluorescence, visited and corresponded with the main centres of study of renal disease and produced a renal biopsy service second to none in the land. His opinion on renal problems was widely sought and he was in great demand as a teacher and lecturer. Contact with active rheumatology departments in Bristol and Bath brought to light more cases of amyloidosis than anyone would have expected. These not only stimulated more research but led to a deep clinical interest in the patients he regularly saw in the 'amyloid clinic' which he organised in conjunction with his clinical colleagues. He was President of the European Amyloidosis Research Symposium held in 1981 and with Professor Paul Bacon edited the report of the proceedings which, published only weeks before his death and providing a masterly summary of the present state of knowledge of the disease, is in itself a fitting memorial to his work on the subject. In all over fifty publications on a variety of pathological topics appeared under his name.

Colin was, however, no blinkered research worker. He saw himself as a general practising histopathologist with some special interests. He had a never-failing infectious enthusiam for all aspects of pathology and never tired of maintaining that a proper understanding of pathology is essential for the conduct of good clinical practice. Every interesting finding had to be shared not only in the laboratory but with the whole clinical staff of the hospital by way of clinico-pathological conferences and contributions to clinical meetings. To this end he amassed thousands of photographs all taken by himself and so methodically filed that he seemed to be able to turn up an example of almost any condition in a few moments. And not only photographs—slides, case reports, reprints and references, similarly carefully catalogued, filled his room from floor to ceiling. He revelled in attempting to solve puzzles and problems and attended every slide seminar and slide presentation meeting that he possibly could, encouraging his juniors to do the same in the belief that this was one of the best forms of continuing education for the histopathologist. He particularly enjoyed the weekly slide discussion meetings organised by the University Pathology Department for the histopathologists in the Bristol area and rarely failed either to produce a helpful comment or express admiration for someone else's erudition. He was a member of many professional societies having a particular affection for the International Academy of Pathology and the Association of Clinical Pathologists on whose education and histopathology committees he served for some years. Never a man to seek high office in the administrative field he nevertheless conscientiously accepted such positions of responsibility as pressure from his peers convinced him he could not decline; as such he served on a number of Regional committees, including the Scientific, Equipment, Laboratory Medicine and Histopathology Committees; of the latter he was the popular and much respected chairman. He frequently represented the Royal College of Pathologists on consultant appointments. He was a Governor of his old school—Clifton. His friends and acquaintances throughout the country were legion and there can hardly be a pathology department in the land where the name of Colin Tribe of Bristol is not known and respected.

Yet few people outside the immediate circle of pathology would have guessed that this genial pipe-smoking friendly and courteous man whom they saw strolling around the hospital looking at trees and flowers was a person of such distinction. He was completely unassuming and without affectation with a genuine love of people for their own sake which made him a most amiable companion.

His enthusiasm for pathology was only matched by that which he lavished on his hobbies. He was a skilled and sensitive wood carver whose creations overflowed from his home to the laboratory, to the hospital and to the homes of his friends. As well as attending woodcarving courses himself he voluntarily arranged classes for the patients of Brentry Hospital. Out of his love for wood grew a great interest in trees. He acquired a piece of land across the water in Wales, known as 'The Plot', where he joyfully laboured creating an arboretum and where, fittingly, his ashes have now been scattered. He was an avid collector of match box labels which flowed into the laboratory from all parts of the earth. He read widely and collected books on all subjects. A great lover of music and singing he founded the hospital choral society, worked to the strains of Mozart from his tape recorder and derived much pleasure from his appointment as deputy tympanist in the hospital orchestra. He travelled widely in his motor caravan. He organised departmental parties and outings and conducted tours of the hospital gardens. He played golf and loved cricket. Readers of this *Journal* will be familiar with his ability to write whimsical jottings about the most unlikely subjects. It was inconceivable that he could ever be idle but he never seemed hurried; there was always time for a chat or a kindly word.

To those who worked with him he was a great colleague and a true friend. Everything he had—his knowledge, his experience, his hobbies—he wished to share with others and the amount of help he gave to so many people is incalculable. The esteem and affection shown to him by everyone of all walks of life at Southmead is unique. Such was his involvement with so many aspects of life in hospital and at home that his going leaves a gap that will never be filled. To his wife, Betty, to whom he was devoted and to his three sons whose welfare was his constant concern, we extend our deepest sympathy.

## Figures and Tables

**Figure f1:**